# Lactoferrin-based food supplements trigger toxin production of enteropathogenic *Bacillus cereus*

**DOI:** 10.3389/fmicb.2023.1284473

**Published:** 2023-11-01

**Authors:** Clara-Sophie Jugert, Andrea Didier, Nadja Jessberger

**Affiliations:** ^1^Department of Veterinary Sciences, Faculty of Veterinary Medicine, Ludwig-Maximilians-Universität München, Oberschleißheim, Germany; ^2^Institute for Food Quality and Food Safety, University of Veterinary Medicine Hannover, Hannover, Germany

**Keywords:** food additive, lactoferrin, *Bacillus cereus*, non-hemolytic enterotoxin, transcriptome, quorum sensing

## Abstract

Lactoferrin is an iron-binding glycoprotein exhibiting antibacterial, antiviral, antifungal, antiparasitic, antiinflammatory, antianaemic and anticarcinogenic properties. While its inhibitory effects against bacterial pathogens are well investigated, little is known about its influence on the production and/or mode of action of bacterial toxins. Thus, the present study aimed to determine the impact of food supplements based on bovine lactoferrin on *Bacillus cereus* enterotoxin production. First, strain-specific growth inhibition of three representative isolates was observed in minimal medium with 1 or 10 mg/mL of a lactoferrin-based food supplement, designated as product no. 1. Growth inhibition did not result from iron deficiency. In contrast to that, all three strains showed increased amounts of enterotoxin component NheB in the supernatant, which corresponded with cytotoxicity. Moreover, lactoferrin product no. 1 enhanced NheB production of further 20 out of 28 *B. cereus* and *Bacillus thuringiensis* strains. These findings again suggested a strain-specific response toward lactoferrin. Product-specific differences also became apparent comparing the influence of further six products on highly responsive strain INRA C3. Highest toxin titres were detected after exposure to products no. 7, 1 and 2, containing no ingredients except pure bovine lactoferrin. INRA C3 was also used to determine the transcriptional response toward lactoferrin exposure *via* RNA sequencing. As control, iron-free medium was also included, which resulted in down-regulation of eight genes, mainly involved in amino acid metabolism, and in up-regulation of 52 genes, mainly involved in iron transport, uptake and utilization. In contrast to that, 153 genes were down-regulated in the presence of lactoferrin, including genes involved in flagellar assembly, motility, chemotaxis and sporulation as well as genes encoding regulatory proteins, transporters, heat and cold shock proteins and virulence factors. Furthermore, 125 genes were up-regulated in the presence of lactoferrin, comprising genes involved in sporulation and germination, nutrient uptake, iron transport and utilization, and resistance. In summary, lactoferrin exposure of *B. cereus* strain-specifically triggers an extensive transcriptional response that considerably exceeds the response toward iron deficiency and, despite down-regulation of various genes belonging to the PlcR-regulon, ultimately leads to an increased level of secreted enterotoxin by a mechanism, which has yet to be elucidated.

## 1. Introduction

*Bacillus cereus* is a Gram-positive, facultative anaerobic, spore forming, ubiquitous soil bacterium. It has gained attention due to its ability to cause two types of foodborne illnesses. On the one hand, the emetic syndrome is caused by the heat-stable cyclic dodecadepsipeptide cereulide leading to nausea and vomiting (Dietrich et al., 2021). On the other hand, enteropathogenic strains are responsible for the diarrhoeal form (Jessberger et al., 2020) by producing three different heat-labile, proteinaceous enterotoxins, which are the single protein CytK (cytotoxin K; Lund et al., 2000) and the two three-component enterotoxin complexes Nhe (non-haemolytic enterotoxin; Lund and Granum, 1996) and Hbl (haemolysin BL; Beecher and Macmillan, 1991). The amount of secreted enterotoxin component NheB provides a good indication for the cytotoxic activity of a *B. cereus* isolate (Moravek et al., 2006). Although several studies have been carried out on this subject, it is still not fully understood under which conditions and in which isolate increased enterotoxin production is triggered. This is due to the complex transcriptional regulation of the enterotoxin genes. The promoter regions of especially the *nhe* and *hbl* operons are unusually long and exhibit binding sites for several transcriptional regulators, which, among others, depend on nutrient and oxygen availability as well as the growth status of the cell (Böhm et al., 2016; Dietrich et al., 2021). Moreover, post-transcriptional and post-translational modifications may play an additional role, which have not yet been clarified in detail (Jessberger et al., 2015). Nevertheless, it has been shown that the bacterium responds with enhanced enterotoxin production to certain environmental factors such as nutrient deprivation and cell density (Gohar et al., 2008), artificial intestinal growth medium (Jessberger et al., 2017) or the presence of mucin (Jessberger et al., 2019). Various food components can also have an impact on toxin production as well as on cytotoxic activity. In a previous study, we were able to show that milk, and especially the milk protein lactoferrin, decreases cytotoxic activity (da Riol et al., 2018).

The approximately 80 kDa iron-binding glycoprotein lactoferrin is a member of the transferrin family (Sienkiewicz et al., 2022). It is most abundant in human and bovine colostrum and milk (Cheng et al., 2008), but it can also be found in further body fluids such as tears, saliva, plasma and semen, as well as in the gastrointestinal, respiratory and reproductive tracts (Superti, 2020). Lactoferrin and its derivatives possess immunomodulatory, antiinflammatory and antioxidant activity, as well as antibacterial, antifungal, antiviral, antiparasitic, antitumour and antianaemic properties (Sienkiewicz et al., 2022; Wu et al., 2023). Furthermore, the molecule can have a positive impact on oral as well as intestinal microbiota (Nakano et al., 2017; Haiwen et al., 2019; Superti, 2020). The antibacterial activity of lactoferrin has already been extensively studied. It exhibits both bacteriostatic and bactericidal effects on various Gram-negative and Gram-positive bacteria. On the one hand, due to its ability to sequester and chelate iron, lactoferrin limits iron availability and thus, bacterial growth (Sienkiewicz et al., 2022). On the other hand, more and more iron-independent mechanisms were discovered such as destabilization of bacterial membranes by binding either to the outer membrane of Gram-negative bacteria and releasing lipopolysaccharides, or to lipoteichoic acids of Gram-positive bacteria (Sienkiewicz et al., 2022). Further, these interactions can enhance the activity of other antibacterials such as lysozyme (Zelechowska et al., 2016). Additional mechanisms are prevention of bacterial adhesion, invasion and biofilm formation, direct interaction with certain bacterial virulence factors, proteolysis of bacterial virulence factors due to serine protease activity, promotion of apoptosis in infected cells, and enhancing bactericidal activity in polymorphonuclear neutrophils (Singh et al., 2002; Ostan et al., 2017; Superti, 2020; Zarzosa-Moreno et al., 2020). Due to such beneficial effects, it is not surprising that a multitude of food supplements based on purified bovine lactoferrin are commercially available. We recently tested a selection of these products (12 commercially available dietary supplements and five purified lactoferrins for research purposes) for their antibacterial activity. Product no. 1, containing no ingredients except pure bovine lactoferrin, strain-specifically inhibited the growth of 112 bacterial isolates. A minor tendency was noted toward stronger growth inhibition of *B. cereus* and *Escherichia coli* compared with *Bacillus thuringiensis*, other *Bacillus*, *Staphylococcus aureus* and *Streptococcus uberis* isolates. However, a general antimicrobial effect against a distinct bacterial species was not observed. Growth inhibition rather depended on the applied strain, product and dose (Jugert et al., 2023). Despite growth inhibition, only little is known about the overall response and especially toxin production of bacterial pathogens after lactoferrin exposure. Several pathogens can counteract iron starvation *via* siderophores, which bind ferric ion with high affinity and transport it into the cells *via* specific membrane receptors. Further specific outer membrane receptors are expressed, which bind and remove iron directly from lactoferrin or transferrin. Thus, many bacteria can use holo-lactoferrin as an iron source (Xiao and Kisaalita, 1997; Wandersman and Stojiljkovic, 2000; Zawadzka et al., 2009). The influence of these processes on toxin production remains largely unclear.

For that reason, the present study aimed to explore the potential effect of lactoferrin-based food supplements on growth and enterotoxin production of *B. cereus*. 31 different strains as well as six different products were compared. Furthermore, the overall transcriptional response of a selected reference strain to lactoferrin exposure was investigated in comparison to iron starvation.

## 2. Materials and methods

### 2.1. Bacterial strains and culture conditions

Twenty-eight *B. cereus* and 3 *B. thuringiensis* strains were used in this study (refer to Supplementary Table S1), including isolates from our own diagnostics laboratories as well as previously published strains (Jessberger et al., 2015; Schwenk et al., 2020). Overnight cultures were incubated for 17 h in CGY medium (casein-glucose-yeast; Jessberger et al., 2015), before bacteria were grown in mMOD (modified, defined medium, which supports good growth and moderate enterotoxin production; Glatz and Goepfert, 1977; Rosenfeld et al., 2005) at 37°C under continuous agitation following previous studies (Jessberger et al., 2019). 20 mL mMOD medium were inoculated with the overnight cultures to OD_600_ = 0.1. Different conditions were compared: mMOD (containing 0.364 μg/mL FeCl_2_), mMOD-Fe (omitting FeCl_2_ as iron source), and mMOD with 1 or 10 mg/mL of different lactoferrin-based food supplements (refer to Table 1). Growth of the three *B. cereus* reference strains F837/76, INRA C3 and NVH 0075–95 depending on lactoferrin-based food supplement no. 1 was determined by measuring the optical density at 600 nm in a photometer (Eppendorf, Hamburg, Germany) once per hour for 12 hours. In a second experiment, the overnight cultures were washed twice in mMOD-Fe before inoculation. Further strains (refer to Supplementary Table S1) were grown for six hours under the same conditions (comparing mMOD and mMOD with 10 mg/mL lactoferrin-based food supplement no. 1), before 2 mL samples were taken. To obtain cell-free supernatants, they were centrifuged (2 min, 10,000 x g, RT). One mM EDTA was added to the supernatant, which was subsequently filtered through 0.2 μm pore-size filters (Merck Millipore, Darmstadt, Germany). 2–3 biological replicates were tested per strain.

**Table 1:** Overview of the commercially available lactoferrin-based dietary supplements used in this study.

Product	Ingredients	Lactoferrin origin	Lactoferrin per capsule
1	Lactoferrin, capsule: hydroxypropylmethylcellulose, gellan	Bovine	400 mg
2	Lactoferrin, capsule: hydroxypropylmethylcellulose	Bovine	250 mg
6	Lactoferrin, glycin, capsule: hydroxypropylmethylcellulose	Bovine	250 mg
7	Lactoferrin, capsule: hydroxypropylmethylcellulose	Bovine	250 mg
8	Lactoferrin, microcrystalline cellulose, L-leucin, calcium salts of orthophosphoric acid, capsule: hydroxypropylmethylcellulose, gellan	Bovine	120 mg
10	Lactoferrin, capsule: hydroxypropylmethylcellulose	Bovine	200 mg
12	Lactoferrin, capsule: hydroxypropylmethylcellulose	Bovine	250 mg

Product no. 1 was used for growth experiments with three representative *B. cereus* strains: F837/76 (haemolysin BL reference strain according to Beecher and Macmillan, 1991), NVH 0075–95 (non-haemolytic enterotoxin reference strain according to Lund and Granum, 1996), and INRA C3 (in-house reference strain). Further, it was used for comparing enterotoxin production of 31 strains (for detailed information see Supplementary Table S1) as well as for transcriptome analyses of strain INRA C3. Products 2, 6, 7, 8, 10 and 12 were used for comparative analyses of enterotoxin production of strain INRA C3. Products are numbered according to Jugert et al. (2023).

### 2.2. Cell line and culture conditions

Vero cells were obtained from ECACC (European Collection of Authenticated Cell Cultures, Salisbury, UK) and cultured in media recommended by the supplier and as described previously (Jessberger et al., 2014).

### 2.3. Determination of iron concentrations

Iron concentrations in the growth media of strain INRA C3 (mMOD, mMOD-Fe, mMOD with 1 mg/mL and mMOD with 10 mg/mL lactoferrin-based food supplement no. 1) were determined in three replicates using the Iron Assay Kit MAK025 (Sigma-Aldrich, Taufkirchen, Germany) as recommended by the supplier.

### 2.4. Enzyme immunoassays (EIAs)

The enterotoxin component NheB was detected in sandwich enzyme immunoassays as described previously (Dietrich et al., 2005; Moravek et al., 2006). Monoclonal antibodies 2B11 (5 μg/mL) and 1E11-HRP (dilution 1:4,000) were used for detection. Tests of different strains were carried out at least in duplicates with three technical replicates each. Microtitre plates were analysed in a Tecan photometer using Magellan 50 software (Tecan, Männedorf, Switzerland). Results are shown as titres, which are defined as the reciprocal of the highest dilutions resulting in an absorbance value of ≥1.0.

### 2.5. Cytotoxicity assays

WST-1 bioassays on Vero cells were performed as previously described (Dietrich et al., 1999, 2005; Jessberger et al., 2014). Supernatants of three *B. cereus* reference strains grown under different conditions (refer to 2.1) were investigated in duplicates with three technical replicates each. Microtitre plates were analysed in a Tecan photometer (Tecan, Männedorf, Switzerland). Dose–response curves as well as 50% inhibitory concentrations were calculated using Magellan 50 software. Data are shown as reciprocal titres.

### 2.6. Transcriptome analyses

One hundred ml mMOD medium were inoculated with strain INRA C3 to OD_600_ = 0.1 and incubated at 37°C for five hours under continuous agitation. The culture was centrifuged (10 min, 4,000 x g, 4°C) and washed twice in cold mMOD-Fe. 50 mL mMOD, mMOD-Fe and mMOD with 10 mg/mL lactoferrin-based food supplement no. 1 were inoculated to OD_600_ = 1 and further incubated for two hours before centrifugation (2 min, 10,000 x g, 4°C). Cell pellets were stored immediately at −80°C. Two biological replicates were prepared per growth condition. Total RNA preparation and on-column DNase digestion were performed using the RNeasy minikit (Qiagen, Hilden, Germany) according to the instructions of the manufacturer. RNA quality was tested *via* spectrophotometer and agarose gel electrophoresis. RNA purity was confirmed *via* PCR for 16S rRNA (Jessberger et al., 2019). RNA was sent to Eurofins Genomics GmbH (Ebersberg, Germany) for initial quality control, rRNA depletion, strand specific random primed cDNA library construction, Illumina sequencing (paired end reads, 2 × 150 bp), mapping of the samples against a reference genome (CP003187.1 *B. cereus* F837/76), and determination of gene expression. On average, 38 million total reads and 35.5 million mapped reads were achieved for each of the six samples. The data presented in the study are deposited in the NCBI’s Gene Expression Omnibus (Edgar et al., 2002) repository, accession number GSE241250.^1^ Data were further processed using Microsoft excel. Only genes with a false discovery rate (FDR) of ≤0.01 and a fold change (logFC) of ≥3 were included in further analyses. Hypothetical proteins were excluded, which accounted for 16.7% (mMOD-Fe vs. mMOD) and 35.8% (mMOD+LF10 vs. mMOD) of all differentially expressed genes. Remaining genes were assigned to functional groups according to the KEGG databases ORTHOLOGY^2^ and - if the latter did not provide any information -BRITE.^3^

### 2.7. Statistical analyses

Data were analyzed using GraphPad Prism version 9.3.1 for Windows, GraphPad Software, San Diego, California USA, www.graphpad.com. Results from iron concentration measurements, EIAs and cytotoxicity assays were compared in relation to the growth conditions “mMOD,” “mMOD-Fe,” “mMOD+LF1” and “mMOD+LF10.” Firstly, data were checked for normal distribution. Secondly, if standard deviations were not significantly different, ordinary one-way ANOVA was performed using Tukey *post hoc* test. If standard deviations were significantly different, Brown-Forsythe and Welch ANOVA tests were performed. When only two conditions were compared, a two-sample t-test was chosen. Significant differences (*p* values <0.05) between growth conditions are indicated as lower case letters in the figures.

## 3. Results

### 3.1. Growth in the presence of lactoferrin

**Figure 1:**
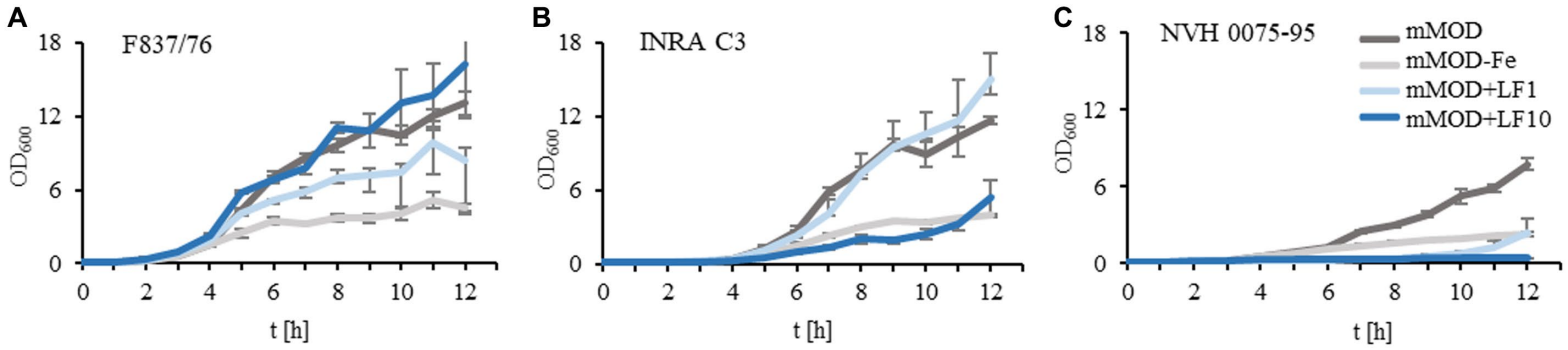
Growth of three *B. cereus* reference strains in the presence of lactoferrin. **(A)** Growth of strain F837/76 in mMOD minimal medium, under iron deficiency (mMOD-Fe) and under addition of 1 mg/mL (mMOD+LF1) and 10 mg/mL (mMOD+LF10) lactoferrin-based food supplement no. 1. **(B)** Growth of strain INRA C3. **(C)** Growth of strain NVH 0075–95.

Previously, we showed large strain-specific differences in the antimicrobial activity of lactoferrin and lactoferrin-based food supplements toward *B. cereus* (Jugert et al., 2023). To investigate this in more detail, growth of three *B. cereus* reference strains in mMOD medium ± iron or lactoferrin-based food supplement no. 1 (compare Table 1) was monitored. Generally, strains F837/76 and INRA C3 grew well in the minimal medium. Growth was clearly reduced under iron deficiency; residual growth might result from traces of iron from the overnight cultures. Addition of 1 mg/mL lactoferrin-based food supplement no. 1 had no influence on strain INRA C3 and only little influence on F837/76. 10 mg/mL clearly inhibited growth of INRA C3, while growth of F837/76 was reinforced (Figures 1A,B). Strain NVH 0075–95, which generally showed the weakest growth, was almost completely inhibited by both 1 and 10 mg/mL (Figure 1C). OD_600_ of all strains incubated under iron deficiency was even further reduced when the overnight cultures were washed twice in mMOD-Fe before incubation. In contrast, growth under all other conditions was comparable (data not shown).

**Figure 2:**
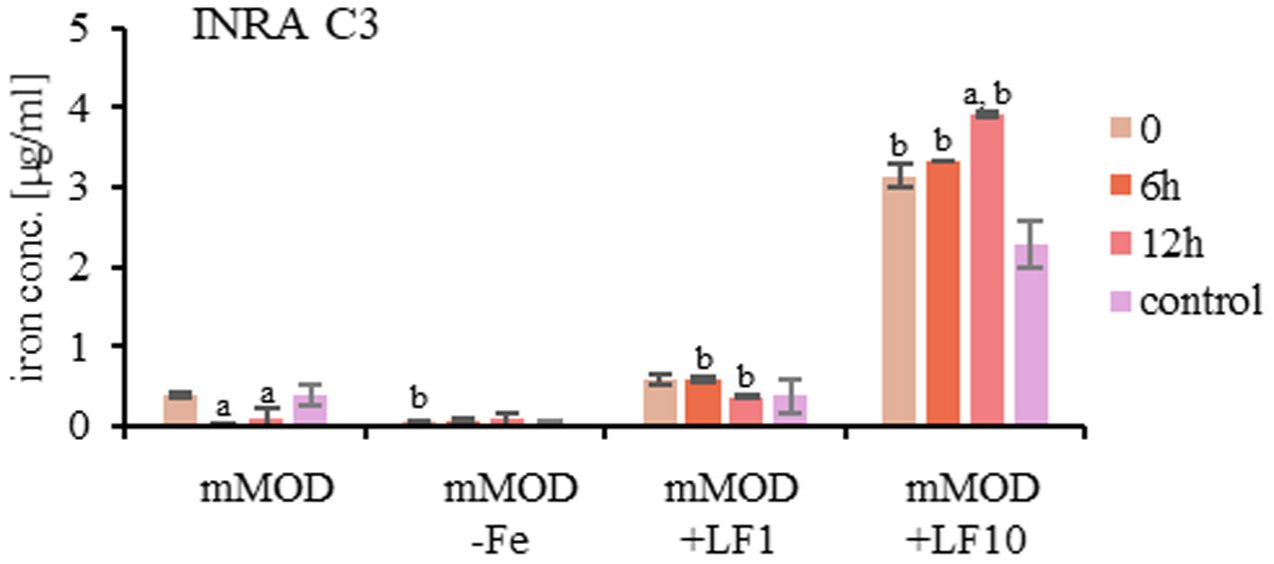
Concentration of freely available iron in the growth media of strain INRA C3. mMOD minimal medium (containing 0.364 μg/mL FeCl_2_), iron-free mMOD-Fe medium and mMOD medium plus 1 mg/mL (mMOD+LF1) and 10 mg/mL (mMOD+LF10) lactoferrin-based food supplement no. 1 were compared. a: significant differences (*p* value <0.05) compared to time point 0 in the same medium. b: significant differences (*p* value <0.05) compared to the mMOD mimimal medium at the same time point.

Simultaneously, the concentration of freely available iron in the medium was determined representatively for strain INRA C3. The mMOD minimal medium itself contains approx. 0.18 μg/mL iron, which was reduced, but not completely used up during bacterial growth. Addition of the lactoferrin product increased the amount of freely available iron, and traces could also be detected in mMOD-Fe (Figure 2).

### 3.2. Strain-specific enhancement of enterotoxin production after lactoferrin exposure

**Figure 3:**
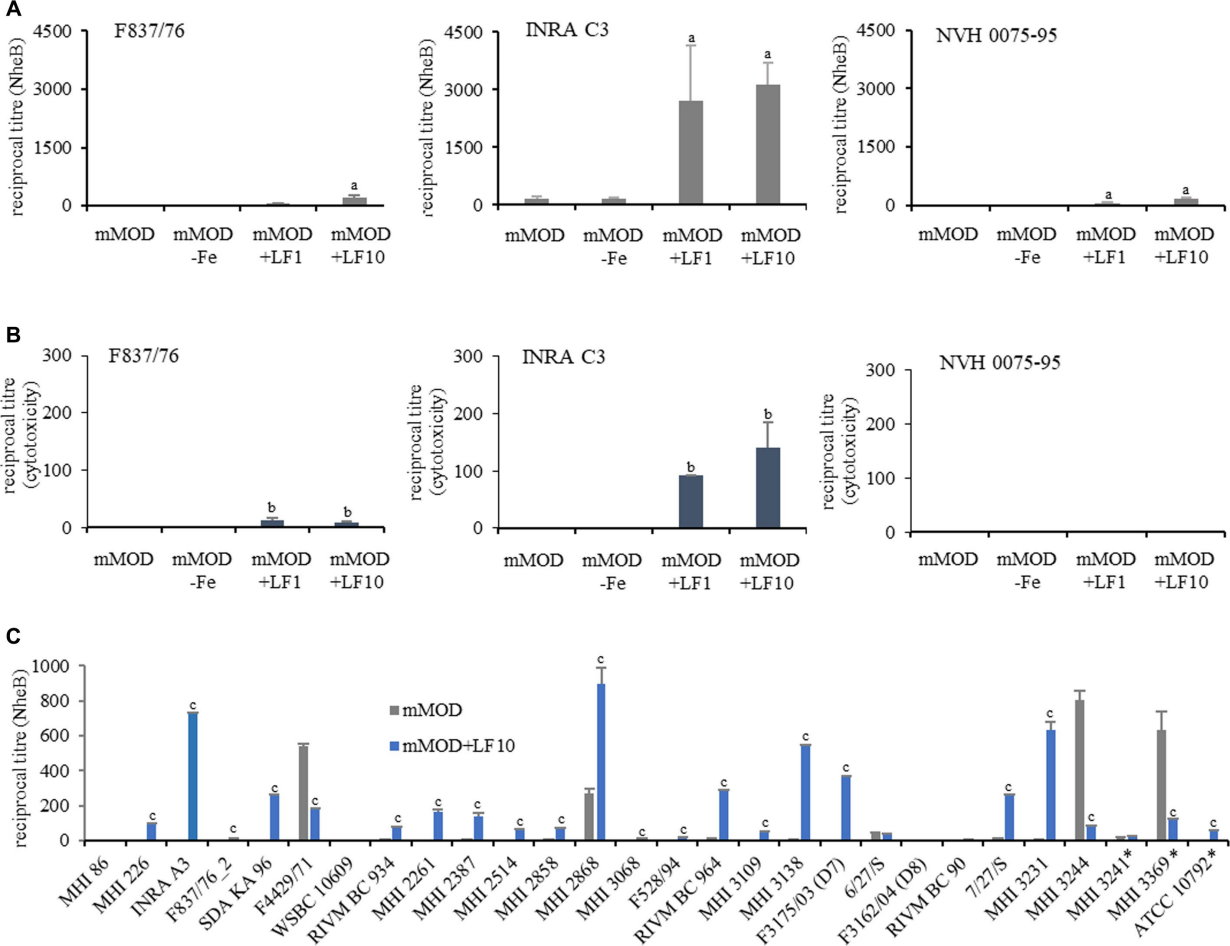
Enterotoxin production in the presence of lactoferrin-based food supplement no. 1. **(A)** Determination of the amounts of enterotoxin component NheB after six hours of growth (compare Figure 1). a: significant differences of reciprocal titres (*p* value <0.05) compared to the pure mMOD minimal medium. **(B)** Corresponding reciprocal titres from WST-1 bioassays on Vero cells, which determine cytotoxicity. b: significant differences of reciprocal titres (p value <0.05) compared to the pure mMOD minimal medium. **(C)** Enterotoxin production (NheB) of further *B. cereus* and *B. thuringiensis* (*) strains. c: significant differences of reciprocal titres (p value <0.05) compared to the pure mMOD minimal medium.

The amount of the secreted enterotoxin component NheB (shown as reciprocal titres) was determined after six hours of growth. In the presence of lactoferrin-based food supplement no. 1, NheB production was clearly enhanced in the three reference strains. Nevertheless, strain INRA C3 showed comparably high titres and F837/76 and NVH 0075–95 comparably low titres under the applied conditions (Figure 3A). Based on this, a comparable picture emerged regarding the cytotoxicity toward Vero cells. INRA C3 showed considerable cytotoxicity titres, F837/76 exhibited very weak cytotoxicity, and for NVH 0075–95 no toxicity was detected at all (Figure 3B).

To clarify if lactoferrin exposure generally leads to enhanced enterotoxin production in *B. cereus* group strains, additional isolates were investigated (*n* = 28, refer to Supplementary Table S1). They were incubated for six hours in mMOD medium ±10 mg/mL lactoferrin-based food supplement no. 1. Out of 25 *B. cereus* strains, 19 showed enhanced enterotoxin production under addition of lactoferrin. Seven of these strains generally produced very low amounts of NheB (with slight enhancement under addition of lactoferrin) under the applied conditions, while three strains showed no enterotoxin production at all, and further three strains showed higher titres in pure mMOD medium. Out of three tested *B. thuringiensis* strains, one showed enhanced NheB production under addition of lactoferrin (Figure 3C).

### 3.3. Product-(in)dependent increase of enterotoxin production

**Figure 4:**
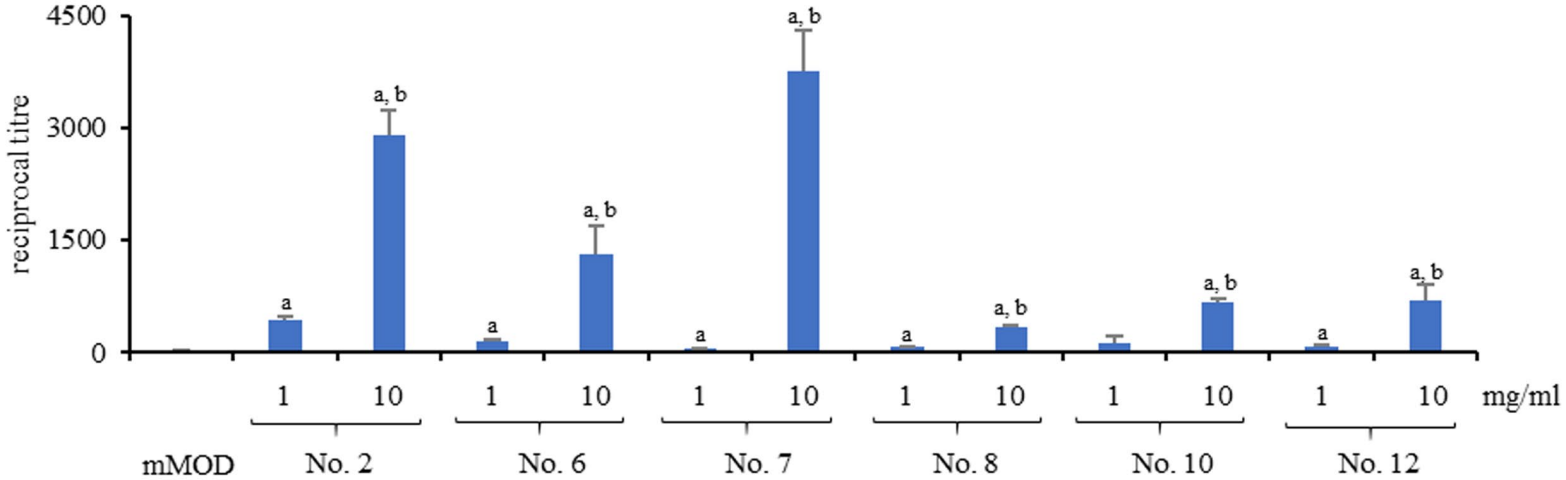
Enterotoxin production of strain INRA C3 after exposure to different lactoferrin-based products. The strain was grown for six hours in mMOD minimal medium under addition of 1 or 10 mg/mL of the products. Product details can be found in Table 1. The culture supernatants were subsequently applied to NheB-specific sandwich EIAs. a: significant differences (*p* value <0.05) compared to the pure mMOD minimal medium. b: significant differences (*p* value <0.05) between 10 and 1 mg/mL.

Reference strain INRA C3, which responded with strong enhancement of enterotoxin production to lactoferrin-based food supplement no. 1, was used to test six further lactoferrin-based dietary supplements (refer to Table 1). Independently of the product, strain INRA C3 showed an increase of enterotoxin production under addition of 1 mg/mL, and a further increase at 10 mg/mL, compared to the mMOD minimal medium. Nevertheless, reciprocal titres for NheB varied depending on the applied product (Figure 4). Highest titres were detected after exposure to products no. 7, no. 1 and no. 2, containing no ingredients except pure bovine lactoferrin.

### 3.4. Transcriptional response of *B. cereus* INRA C3 to lactoferrin exposure

**Figure 5:**
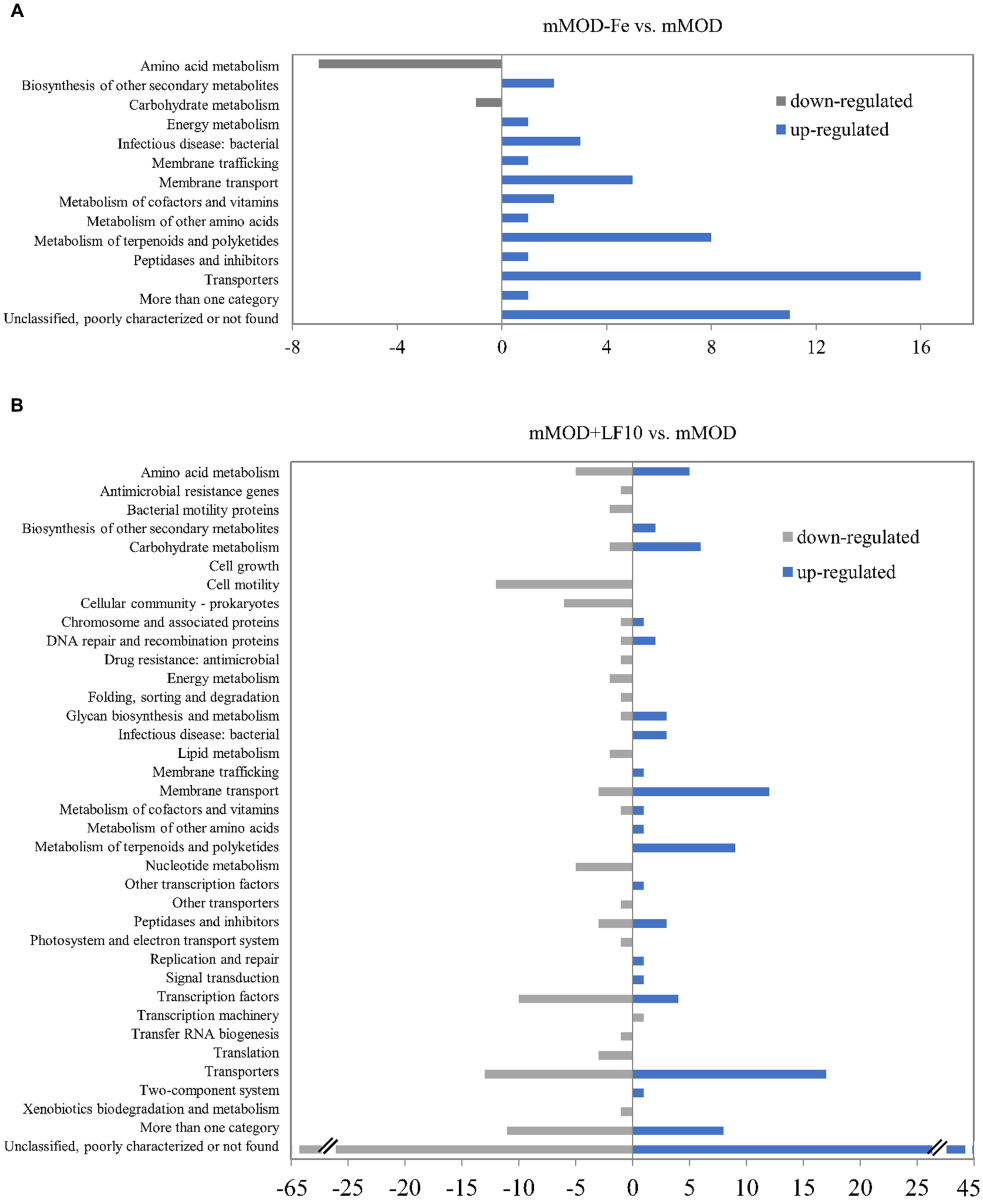
Differential gene regulation in *B. cereus* strain INRA C3 after two hours of incubation in mMOD, mMOD-Fe and mMOD with 10 mg/mL lactoferrin-based food supplement no. 1. Genes with a false discovery rate (FDR) of ≤0.01 and a fold change (logFC) of ≥3 (excluding hypothetical proteins) were assigned to functional groups according to the KEGG databases ORTHOLOGY (https://www.genome.jp/kegg/ko.html) and BRITE (https://www.genome.jp/kegg/brite.html). **(A)** Eight genes were down-regulated under iron starvation (mMOD-Fe; grey bars) compared to mMOD, while 52 genes were up-regulated (blue bars). **(B)** 153 genes were down-regulated in the presence of lactoferrin (mMOD+LF10; grey bars) compared to mMOD, while 125 genes were up-regulated (blue bars). Details are shown in Supplementary Tables S2, S3.

To gain more detailed insights into the response of *B. cereus* to lactoferrin exposure, total transcriptome analyses of strain INRA C3 were performed *via* RNA sequencing. Gene expression after two hours of incubation in mMOD with 10 mg/mL lactoferrin-based food supplement no. 1, which resulted earlier in reduced growth, but enhanced enterotoxin production of this strain, was compared to the pure minimal medium. In addition and as a control, gene expression in the iron-free mMOD-Fe medium was also compared to the pure minimal medium. Under the applied conditions, eight genes, mainly involved in amino acid metabolism, were down-regulated under iron starvation (mMOD-Fe), while 52 genes, mainly involved in iron transport, uptake and utilization, were up-regulated. “Transporters” was the most represented functional group (compare Figure 5A and Supplementary Table S2). In comparison to that, a higher number of genes was differentially regulated in the presence of lactoferrin. 153 genes were down-regulated, among others including genes involved in flagellar assembly, motility, chemotaxis and sporulation as well as genes encoding regulatory proteins, transporters, heat and cold shock proteins and - most interestingly - phosphatidylinositol-specific phospholipase C, the PlcR-activating protein PapR, and the *hbl* operon. On the other hand, 125 genes were up-regulated in the presence of lactoferrin, among others again comprising genes involved in sporulation and germination, but also genes generally involved in nutrient uptake, in iron transport and utilization, and resistance toward polymyxin, vancomycin and heavy metals (compare Figure 5B and Supplementary Table S3).

## 4. Discussion

With the exception of iron acquisition, relatively little focus has been placed on the reaction of bacterial pathogens, especially toxin-forming organisms, toward lactoferrin exposure. In an earlier review, it was suggested that the sequestration of iron inhibits bacterial growth, limits the use of this nutrient and down-regulates virulence factor expression (Garcia-Montoya et al., 2012). Indeed, a previous study with a similar methodological approach to the present one revealed a decrease of active, cell-free Stx2, but not Stx1 toxin in EHEC cultures depending on the concentration of applied bovine lactoferrin. The molecule also significantly reduced cytotoxicity of colonizing bacteria toward Vero cells. These effects were partly attributed to degradation of Stx2 by lactoferrin (Kieckens et al., 2017). On the contrary, the present study shows, despite strain-specific growth inhibition by lactoferrin, increased enterotoxin (NheB) production of 23 out of 31 tested *B. cereus* and *B. thuringiensis* strains. Results of three selected reference strains suggest that there is not necessarily a connection between growth inhibition by lactoferrin and increased enterotoxin secretion. Lactoferrin concentrations were selected based on its physiological concentrations, which are described as up to 7 mg/mL in milk and up to 2 mg/mL in mucus and mucosa. However, these concentrations can be highly variable (Ling and Schryvers, 2006). Regarding utilization of lactoferrin by *B. cereus*, there are contradictory findings in the literature. Strain ATCC 14579 has been shown to use hemoglobin, haem and ferritin, but not transferrin and lactoferrin as iron source (Daou et al., 2009). Sato et al. concluded that *B. cereus* is not able to use transferrin or lactoferrin (Sato et al., 1999a,b), while Hayrapetyan et al. (2016) noted utilization of transferrin, ferritin and lactoferrin by six, three and two out of 22 tested strains, respectively. In a recent study, we determined that growth inhibition by lactoferrin varies strain-specifically, not only, but especially within the *B. cereus* species. Here, strain F837/76 showed itself to be particularly resistant to various lactoferrin-based food supplements (Jugert et al., 2023). Due to its increased growth under high lactoferrin concentrations in the present study, it is likely that F837/76 belongs to the rather rare strains that can use lactoferrin as a source of iron and growth. Highly responsive strain INRA C3 showed highest toxin production in the present study when exposed to lactoferrin-based food supplements containing only pure bovine lactoferrin; product no. 8, which resulted in the lowest enhancing effect, additionally consists of microcrystalline cellulose, L-leucin and calcium salts of orthophosphoric acid (compare Table 1). Nevertheless, further studies are needed to investigate the individual and combined effects of each ingredient on enterotoxin production.

The majority of publications focuses on bacterial counteractions of lactoferrin-induced iron starvation and the identification of lactoferrin-binding proteins. These proteins and/or mechanisms have already been identified in *Neisseria* spp., *Moraxella* spp., *Helicobacter pylori*, *S. aureus*, *S. uberis* and others (Ling and Schryvers, 2006), *Pseudomonas aeruginosa* (Xiao and Kisaalita, 1997) and *B. cereus* (Zawadzka et al., 2009). In contrast to that, the present study demonstrates that growth inhibition as well as enhanced enterotoxin production of the tested *B. cereus* strains does not result from iron sequestration by lactoferrin and thus, iron deficiency in the growth medium. This was also confirmed by the transcriptional response of strain INRA C3, which was considerably more pronounced under lactoferrin exposure than under iron deficiency. Under both conditions, genes involved in iron transport and metabolism showed increased expression. These results are largely consistent with a previous study in which, following artificially induced iron deficiency, upregulation of predicted iron transporters was monitored in *B. cereus* strain ATCC 10987 (Hayrapetyan et al., 2016). Under lactoferrin exposure, however, additionally genes involved in sporulation and germination, nutrient uptake, resistance, etc. were up-regulated. Most interestingly, genes encoding parts of the *hbl* operon were down-regulated under lactoferrin exposure. This confirms the results of enzyme immunoassays against the toxin component Hbl L2, where we could not detect a clearly enhanced or attenuated toxin production compared to the mMOD minimal medium, neither under iron starvation nor under addition of lactoferrin (data not shown). Genes encoding further virulence factors, such as bacillolysin/neutral protease (Sidler et al., 1986) or phosphatidylinositol-specific phospholipase C (PI-PLC) (Kuppe et al., 1989), were also down-regulated. Interestingly, we also detected decreased expression of the gene encoding PlcR-activating protein PapR after lactoferrin exposure. In *B. cereus*, gene expression of the majority of secreted proteins including *nhe*, *hbl*, *cytK*, *smase*, *plcA* (PI-PLC), *plcB* (PC-PLC), and *inhA2* is regulated *via* the PlcR-PapR quorum-sensing system (Dietrich et al., 2021; Prince and Kovac, 2022). PlcR is a pleiotropic transcriptional regulator, which activates gene transcription at the onset of the stationary growth phase (Gohar et al., 2008). The signaling peptide PapR is exported, processed *via* neutral protease NprB and re-imported as heptapeptide PapR7 *via* the oligopeptide permease OppABCDF. At high cell densities, PapR is accumulated as signaling molecule inside the cell and activates PlcR (Gominet et al., 2001; Grenha et al., 2013; Dietrich et al., 2021). The reduced expression of enterotoxin genes and other virulence factors after lactoferrin exposure could be explained by the downregulation of the PlcR-PapR quorum sensing system. This system is associated with cytotoxicity, responsible for overcoming obstacles in the host gut and suggested to be an excellent target for antivirulence compounds (Prince and Kovac, 2022). Lactoferrin seems to be one of these compounds.

In accordance with that, it has to be noted that no enhanced expression of the *nhe* operon was detected under the applied conditions. On the contrary, if less strict selection criteria were used with respect to logFC (fold change), a slight down-regulation of the *nheB* gene could be observed (data not shown). Nevertheless, we measured increased amounts of secreted enterotoxin in the supernatant (see above). However, these observations are to no extent contradictory. It has already been shown in previous studies that enterotoxin gene transcription and the level of toxin actually secreted by *B. cereus* are not necessarily correlated (Jessberger et al., 2015, 2017). Instead, enterotoxin production is determined by the combination of highly complex, partially interrelated processes of gene transcription, posttranscriptional and posttranslational modification, and protein secretion, which have not yet been fully understood (Dietrich et al., 2021). Lactoferrin may also influence additional unknown activities, such as processing or degradation of the enterotoxins by extracellular bacterial proteases. Furthermore, an intrinsic protease activity of lactoferrin has been described, thus preventing colonization of mucosal surfaces by *Haemophilus influenzae* or inhibiting adhesion and invasion by degrading effector proteins of the type III secretion systems in *E. coli* and *Shigella flexneri* (Ling and Schryvers, 2006). Lactoferrin appears to exhibit serine protease activity, cleaving arginine-rich sequences of target proteins (Hendrixson et al., 2003; Ochoa et al., 2003). Hendrixson et al. (2003) further postulated that the molecule may cleave arginine-rich sequences in many microbial virulence factors, thus contributing to its long-recognized antimicrobial properties. However, the *B. cereus* enterotoxins are most likely not affected by this, as they contain only a few arginine residues (for example four arginine residues in F837/76 NheB) and rather no arginine-rich sequences at all. Nevertheless, experimental proof of this has not yet been provided. In the concentrations applied in the present study, lactoferrin itself did not interact with the antibodies used in enzyme immunoassays or with Vero cells in cytotoxicity assays, as tested by a number of negative controls.

## 5. Conclusion

In spite of the various beneficial effects of lactoferrin, especially with regard to its antibacterial properties, it is important to remember that pathogens are able to take defensive measures. In the case of enteropathogenic *B. cereus*, these relate not only to the ability of certain isolates to use lactoferrin as an iron source for growth, but also, to a particular extent, to increased enterotoxin production after lactoferrin exposure. Thus, the present study adds a new perspective on the extensive use of lactoferrin in human medicine and as popular food supplement.

## Data availability statement

The datasets presented in this study can be found in online repositories. The names of the repository/repositories and accession number(s) can be found at: https://www.ncbi.nlm.nih.gov/geo/, GSE241250.

## Ethics statement

Ethical approval was not required for the studies on animals in accordance with the local legislation and institutional requirements because only commercially available established cell lines were used.

## Author contributions

C-SJ: Data curation, Formal Analysis, Investigation, Methodology, Writing – original draft. AD: Conceptualization, Supervision, Writing – review and editing. NJ: Conceptualization, Data curation, Formal analysis, Funding acquisition, Supervision, Writing – original draft.

## Footnotes

^1^ https://www.ncbi.nlm.nih.gov/geo/query/acc.cgi?acc=GSE241250

^2^ https://www.genome.jp/kegg/ko.html

^3^ https://www.genome.jp/kegg/brite.html
