# Supplementary material for: Lactoferrin-based food supplements trigger toxin production of enteropathogenic Bacillus cereus
Source: Front Microbiol. 2023 Nov 1;14:1284473. doi: 10.3389/fmicb.2023.1284473 (PMC10646309; doi:10.3389/fmicb.2023.1284473)
Supplement: Supplementary file 1 [file Data_Sheet_1.docx]

Supplementary Material

**Lactoferrin-based food supplements trigger toxin production**

**of enteropathogenic *Bacillus cereus***

Clara-Sophie Jugert, Andrea Didier, Nadja Jessberger

**Table S1.** **Overview of the bacterial species and strains used in this study.** *: Previously published strains according to Jeßberger et al. (2015). **: Previously published strains according to Schwenk et al. (2020). ***: Previously published strains according to Jugert et al. (2023). F837/76: Hbl reference strain according to Beecher & Macmillan (1991). NVH 0075-95: Nhe reference strain according to Lund & Granum (1996). INRA C3: In house reference strain. ATCC: Purchased from the American Type Culture Collection.

| **Isolate** | **Origin** |
| --- | --- |
| ***Bacillus cereus*** | |
| F837/76* | Postoperative infection |
| INRA C3* | Pasteurized carrots |
| NVH 0075-95* | Vegetable stew, food poisoning |
| MHI 86* | Infant food |
| MHI 226* | Milk product |
| INRA A3* | Starch |
| F837/76_2*** | Postoperative infection |
| SDA KA96* | Raw milk |
| F4429/71* | Vanilla pudding |
| WSBC 10609 | Vomit |
| RIVM BC 934* | Salad |
| MHI 2261*** | Belgium, vomit, emetic |
| MHI 2387*** | Vitamin paste |
| MHI 2514 | Mushroom cream sauce |
| MHI 2858 | Noodle salad |
| MHI 2868 | Milk sample |
| MHI 3068 | Potato salad |
| F528/94* | Rice dish, food poisoning |
| RIVM BC 964* | Kebab |
| MHI 3109*** | Kebab, food poisoning with vomiting |
| MHI 3138*** | Fried noodles, emetic toxin |
| F3175/03 (D7)* | Human faeces |
| 6/27/S* | Human faeces |
| F3162/04 (D8)* | Human faeces |
| RIVM BC 90* | Human faeces |
| 7/27/S* | Human faeces |
| MHI 3231*** | Hazelnut ice cream |
| MHI 3244 | Vegetable casserole |
| ***Bacillus thuringiensis*** | |
| MHI 3241** | ssp. *tenebrionis*, biopesticide |
| MHI 3369** | ssp. *aizawai*, biopesticide |
| ATCC 10792** | - |

**Table S2. Differentially expressed genes of *B. cereus* strain INRA C3 after two hours of growth in mMOD-Fe (iron-free) medium compared to mMOD medium.** Only genes with a false discovery rate (FDR) of ≤ 0.01 and a fold change (logFC) of ≥ 3 were included. Hypothetical proteins were excluded (0 down-regulated, 12 up-regulated under iron starvation). Eight genes were down-regulated under iron starvation (-), while 52 genes were up-regulated (no algebraic sign). Genes were assigned to functional groups according to the KEGG databases ORTHOLOGY (https://www.genome.jp/kegg/ko.html) and BRITE (https://www.genome.jp/kegg/ brite.html).

| **#Accession** | **Gene** | **logFC** | **Functional group** |
| --- | --- | --- | --- |
| Bcf_06210 | Anthranilate synthase, amidotransferase component | -6.29 | Amino acid metabolism |
| Bcf_06220 | Indole-3-glycerol phosphate synthase | -6.20 | Amino acid metabolism |
| Bcf_06215 | Anthranilate phosphoribosyltransferase | -6.04 | Amino acid metabolism |
| Bcf_06230 | Tryptophan synthase subunit beta | -5.81 | Amino acid metabolism |
| Bcf_06225 | Phosphoribosylanthranilate isomerase | -5.74 | Amino acid metabolism |
| Bcf_06235 | Tryptophan synthase subunit alpha | -5.16 | Amino acid metabolism |
| Bcf_06205 | Anthranilate synthase, aminase component | -4.71 | Amino acid metabolism |
| Bcf_01850 | Gamma-aminobutyrate:alpha-ketoglutarate aminotransferase | -3.07 | Carbohydrate metabolism |
| Bcf_25910 | ABC transporter ATP-binding protein/permease | 3.03 | Transporters |
| Bcf_16190 | AraC family transcriptional regulator | 3.11 | Not found |
| Bcf_09570 | Cytochrome d ubiquinol oxidase subunit I | 3.16 | Energy metabolism |
| Bcf_02005 | Excisionase family DNA binding domain | 3.20 | Not found |
| Bcf_01980 | Fe3+-siderophore ABC transporter permease 2 component | 3.57 | Membrane transport |
| Bcf_17155 | Fe3+-siderophore ABC transporter permease 2 component | 3.59 | Membrane transport |
| Bcf_21995 | Substrate-binding family protein | 3.75 | Not found |
| Bcf_17150 | Fe-bacillibactin uptake system FeuA | 3.88 | Transporters |
| Bcf_01985 | Fe3+-siderophore ABC transporter permease | 3.94 | Transporters |
| Bcf_22670 | Iron compound ABC transporter permease | 4.00 | Membrane transport |
| Bcf_02835 | Internalin | 4.13 | Infectious disease: bacterial |
| Bcf_22665 | Ferrichrome-binding periplasmic protein | 4.29 | Transporters |
| Bcf_09760 | Long-chain-fatty-acid--CoA ligase | 4.37 | Metabolism of terpenoids and polyketides |
| Bcf_01990 | Ferrichrome-binding periplasmic protein | 4.54 | Transporters |
| Bcf_25550 | Petrobactin ABC transporter substrate-binding protein | 4.59 | Not found |
| Bcf_05520 | S-layer protein | 4.76 | Unclassified: Structural proteins |
| Bcf_17160 | Fe3+-siderophore ABC transporter permease | 4.86 | Transporters |
| Bcf_11780 | 2,3-dihydro-2,3-dihydroxybenzoate dehydrogenase | 4.89 | Metabolism of terpenoids and polyketides |
| Bcf_04035 | Putative nitroreductase family protein | 4.90 | Metabolism of cofactors and vitamins / Signal transduction |
| Bcf_09765 | Acyl carrier protein associated with anthrachelin biosynthesis | 5.02 | Metabolism of terpenoids and polyketides |
| Bcf_09755 | Anthrachelin biosynthesis protein AsbB / Siderophore synthetase superfamily, group C / Siderophore synthetase component, ligase | 5.03 | Metabolism of terpenoids and polyketides |
| Bcf_01995 | Thioredoxin reductase | 5.07 | Metabolism of other amino acids |
| Bcf_14575 | Antibiotic biosynthesis monooxygenase domain- containing protein | 5.16 | Biosynthesis of other secondary metabolites |
| Bcf_09750 | Anthrachelin biosynthesis protein AsbA / Siderophore synthetase superfamily, group A / Siderophore synthetase large component, acetyltransferase | 5.85 | Metabolism of terpenoids and polyketides |
| Bcf_22745 | Heme transporter analogous to IsdDEF, ATP- binding protein | 6.51 | Transporters |
| Bcf_22750 | Heme transporter IsdDEF, permease component IsdF | 6.58 | Transporters |
| Bcf_18510 | Trilactone hydrolase (bacillibactin) siderophore | 6.70 | Not Included in Pathway or Brite |
| Bcf_22740 | NPQTN specific sortase B | 6.87 | Peptidases and inhibitors |
| Bcf_18515 | Fe-bacillibactin uptake system FeuD | 7.01 | Transporters |
| Bcf_22755 | Heme transporter IsdDEF, lipoprotein IsdE | 7.05 | Membrane transport |
| Bcf_11815 | 4'-phosphopantetheinyl transferase | 7.17 | Metabolism of cofactors and vitamins |
| Bcf_11785 | Isochorismate synthase | 7.18 | Metabolism of cofactors and vitamins |
| Bcf_11795 | Isochorismatase | 7.52 | Metabolism of terpenoids and polyketides |
| Bcf_11800 | Siderophore biosynthesis non-ribosomal peptide synthetase / Bacillibactin synthetase component F | 7.57 | Metabolism of terpenoids and polyketides |
| Bcf_18525 | Fe-bacillibactin uptake system FeuB | 7.65 | Transporters |
| Bcf_11810 | Polymyxin transporter PmxC | 7.67 | Not found |
| Bcf_11805 | Polymyxin synthetase PmxB | 7.74 | Biosynthesis of other secondary metabolites |
| Bcf_18520 | Fe-bacillibactin uptake system FeuC | 7.82 | Transporters |
| Bcf_11790 | 2,3-dihydroxybenzoate-AMP ligase | 7.95 | Metabolism of terpenoids and polyketides |
| Bcf_21715 | Ankyrin repeat domain protein | 7.99 | Membrane trafficking |
| Bcf_16240 | Internalin | 8.33 | Infectious disease: bacterial |
| Bcf_03370 | Oligopeptide ABC transporter substrate-binding protein OppA | 8.72 | Membrane transport |
| Bcf_21720 | Iron compound ABC transporter ATP-binding protein | 8.73 | Transporters |
| Bcf_22760 | Cell surface protein IsdA | 9.09 | Transporters |
| Bcf_18535 | Fe-bacillibactin uptake system FeuA | 9.15 | Transporters |
| Bcf_05665 | S-layer protein | 9.61 | Unclassified: Structural proteins |
| Bcf_06735 | Internalin | 9.87 | Infectious disease: bacterial |
| Bcf_22765 | NPQTN cell wall anchored protein IsdC | 10.19 | Not found |
| Bcf_21725 | Fe3+-siderophore ABC transporter permease | 10.46 | Transporters |
| Bcf_21730 | Heme transporter IsdDEF, lipoprotein IsdE | 10.94 | Transporters |
| Bcf_06965 | Flavodoxin | 11.84 | Unclassified: Energy metabolism |
| Bcf_17445 | Flavodoxin | 12.27 | Unclassified: Energy metabolism / Quorum sensing / Drug resistance |

**Table S3. Differentially expressed genes of *B. cereus* strain INRA C3 after two hours of growth in mMOD plus 10 mg/ml lactoferrin-based food supplement no. 1 compared to mMOD medium.** Only genes with a false discovery rate (FDR) of ≤ 0.01 and a fold change (logFC) of ≥ 3 were included. Hypothetical proteins were excluded (121 down-regulated, 34 up-regulated in the presence of lactoferrin). 153 genes were down-regulated in the presence of lactoferrin (-), while 125 genes were up-regulated (no algebraic sign). Genes were assigned to functional groups according to the KEGG databases ORTHOLOGY (https://www.genome.jp/kegg/ko.html) and BRITE (https://www.genome.jp/kegg/brite.html).

| **#Accession** | **Gene** | **logFC** | **Functional group** |
| --- | --- | --- | --- |
| Bcf_14055 | Spore coat protein W | -9.47 | Unclassified: Metabolism |
| Bcf_10600 | Exosporium protein G | -9.29 | Unclassified: Cell growth |
| Bcf_22835 | Small, acid-soluble spore protein | -8.60 | Unclassified: Cell growth |
| Bcf_08195 | Stage 0 sporulation regulatory protein | -7.83 | Signal transduction / Cellular community - prokaryotes |
| Bcf_25295 | Methyl-accepting chemotaxis protein | -7.45 | Signal transduction / Cell motility |
| Bcf_25875 | DNA-binding protein | -7.38 | Transcription factors |
| Bcf_14050 | Spore coat protein X | -7.34 | Unclassified: Cell growth |
| Bcf_04440 | Alpha/beta family small acid-soluble spore protein | -6.77 | Unclassified: Cell growth |
| Bcf_08380 | Flagellin protein FlaA | -6.69 | Signal transduction / Cell motility / Flagellar assembly |
| Bcf_24755 | Stage 0 sporulation regulatory protein | -6.18 | Signal transduction / Cellular community - prokaryotes |
| Bcf_17945 | Small, acid-soluble spore protein O | -6.03 | Unclassified: Cell growth |
| Bcf_08440 | Flagellar basal-body rod protein FlgG | -5.87 | Cell motility |
| Bcf_11760 | ArsR family transcriptional regulator | -5.73 | Transcription factors |
| Bcf_26175 | Arsenic efflux pump protein | -5.57 | Transporters |
| Bcf_21595 | 50S ribosomal protein L33 | -5.56 | Translation |
| Bcf_02210 | PBSX family transcriptional regulator | -5.45 | Transcription factors |
| Bcf_08415 | Flagellar biosynthesis protein FliQ | -5.31 | Cell motility |
| Bcf_11840 | Alkaline serine protease, subtilase family | -5.30 | Cellular community - prokaryotes |
| Bcf_04180 | ABC transporter permease | -5.23 | Membrane transport |
| Bcf_06025 | Adenylate cyclase | -5.22 | Not found |
| Bcf_02200 | TetR family transcriptional regulator | -5.21 | Transcription factors |
| Bcf_08710 | Transcriptional regulator | -5.20 | Not found |
| Bcf_25480 | Endonuclease/exonuclease/phosphatase family protein | -5.16 | Not found |
| Bcf_07935 | Xanthine phosphoribosyltransferase | -5.14 | Nucleotide metabolism |
| Bcf_03040 | Cadmium efflux system accessory protein | -5.13 | Not found |
| Bcf_24590 | Alpha/beta fold family hydrolase | -5.11 | Not found |
| Bcf_23310 | Alpha/beta family small acid-soluble spore protein | -5.10 | Unclassified: Cell growth |
| Bcf_18650 | Phosphatidylinositol-specific phospholipase C | -4.99 | Carbohydrate metabolism |
| Bcf_26115 | Integral membrane protein | -4.94 | Unclassified: Structural proteins |
| Bcf_12165 | Exosporium protein J | -4.93 | Not found |
| Bcf_02565 | Gamma-type small acid-soluble spore protein | -4.91 | Unclassified: Cell growth |
| Bcf_07360 | Phosphate transport regulator | -4.90 | Unclassified: Cell growth |
| Bcf_09780 | Alpha/beta family small acid-soluble spore protein | -4.84 | Poorly characterized |
| Bcf_05450 | Transcriptional repressor, BlaI/MecI family | -4.79 | Drug resistance: antimicrobial |
| Bcf_26855 | PlcR activating protein PapR, quorum-sensing effector | -4.78 | Cellular community - prokaryotes |
| Bcf_08355 | Chemotaxis protein CheV | -4.68 | Signal transduction / Cell motility |
| Bcf_15215 | Alpha/beta family small acid-soluble spore protein | -4.62 | Unclassified: Cell growth |
| Bcf_22445 | Major facilitator family transporter | -4.62 | Transporters |
| Bcf_25405 | Sodium/alanine symporter family protein | -4.60 | Unclassified: Cell growth |
| Bcf_10550 | Molybdenum cofactor biosynthesis protein MoaD | -4.60 | Metabolism of cofactors and vitamins |
| Bcf_26230 | Diguanylate cyclase/phosphodiesterase domain 1 | -4.58 | Nucleotide metabolism |
| Bcf_12185 | GTP pyrophosphokinase | -4.57 | Nucleotide metabolism |
| Bcf_23765 | HAD superfamily hydrolase | -4.55 | Poorly characterized |
| Bcf_26110 | Proton/sodium-glutamate symport protein | -4.55 | Transporters |
| Bcf_08070 | Cold shock protein CspB | -4.42 | Unclassified: Transcription |
| Bcf_15530 | NADH dehydrogenase | -4.38 | Energy metabolism |
| Bcf_08310 | Flagellar motor switch protein FliG | -4.33 | Cell motility / Flagellar assembly |
| Bcf_03135 | Di-/tripeptide transporter | -4.29 | Unclassified: Transport |
| Bcf_13845 | Carboxymuconolactone decarboxylase | -4.26 | Xenobiotics biodegradation and metabolism |
| Bcf_08220 | Chemotaxis regulator | -4.26 | Signal transduction / Cell motility |
| Bcf_02475 | UDP-glucose/GDP-mannose dehydrogenase family | -4.25 | Carbohydrate metabolism |
| Bcf_25375 | Nucleoside permease NupC | -4.22 | Membrane transport |
| Bcf_16075 | DNA polymerase IV | -4.19 | DNA repair and recombination proteins |
| Bcf_27135 | Integral membrane protein | -4.16 | Poorly characterized |
| Bcf_25400 | Sodium/alanine symporter family protein | -4.14 | Unclassified: Cell growth |
| Bcf_26070 | Spermidine synthase | -4.12 | Amino acid metabolism |
| Bcf_25380 | Nucleoside permease NupC | -4.11 | Membrane transport |
| Bcf_08395 | Flagellar motor switch protein FliM | -4.09 | Cell motility |
| Bcf_08300 | Flagellar hook-basal body complex protein FliE | -4.07 | Cell motility |
| Bcf_05895 | Putative repressor of comG operon | -4.07 | Not found |
| Bcf_15285 | HblB protein | -4.06 | Cellular community - prokaryotes |
| Bcf_06640 | Alpha/beta family small acid-soluble spore protein | -4.05 | Unclassified: Cell growth |
| Bcf_00620 | 50S ribosomal protein L10 | -4.04 | Translation |
| Bcf_24875 | Lipoprotein | -4.04 | Not found |
| Bcf_08050 | Queuosine-regulated ECF transporter substrate-specific protein QueT | -4.04 | Not found |
| Bcf_16785 | Serine transporter | -4.03 | Transporters |
| Bcf_22585 | Flagellar motor rotation protein MotA | -4.02 | Signal transduction / Cell motility |
| Bcf_25260 | Protein erfK/srfK like precursor | -4.00 | Peptidases and inhibitors |
| Bcf_14060 | Spore coat protein X | -3.99 | Unclassified: Cell growth |
| Bcf_06900 | Leucine-responsive regulatory protein | -3.98 | Transcription factors |
| Bcf_09890 | Methyl-accepting chemotaxis protein | -3.97 | Signal transduction / Cell motility |
| Bcf_03515 | Paramyx_RNA_pol, Paramyxovirus RNA dependent RNA polymerase | -3.96 | Not found |
| Bcf_08410 | Flagellar biosynthesis protein FliP | -3.96 | Cell motility |
| Bcf_11340 | Heat shock protein, Hsp20 family | -3.95 | Folding, sorting and degradation |
| Bcf_01130 | Lipoprotein | -3.94 | Not found |
| Bcf_10025 | Exosporium protein B | -3.86 | Unclassified: Cell growth |
| Bcf_02240 | LPXTG-motif cell wall anchor domain-containing protein | -3.85 | Not found |
| Bcf_02310 | Drug/metabolite transporter permease | -3.84 | Other transporters |
| Bcf_13455 | Spore cortex-lytic enzyme, lytic transglycosylase SleB | -3.83 | Unclassified: Metabolism |
| Bcf_08400 | Flagellar motor switch protein FliN | -3.83 | Cell motility |
| Bcf_26970 | MerR family transcriptional regulator | -3.78 | Transcription factors |
| Bcf_08430 | Flagellar biosynthesis protein FlhA | -3.77 | Cell motility |
| Bcf_17315 | Response regulator | -3.76 | Cellular community - prokaryotes |
| Bcf_15295 | Hemolysin BL lytic component L2 | -3.76 | Cellular community - prokaryotes |
| Bcf_00840 | tRNA pseudouridine synthase A | -3.76 | Transfer RNA biogenesis |
| Bcf_08405 | Flagellar motor switch protein FliN | -3.76 | Cell motility |
| Bcf_08255 | UDP-N-acetylenolpyruvoylglucosamine reductase | -3.75 | Poorly characterized |
| Bcf_02305 | Lead, cadmium, zinc and mercury transporting ATPase | -3.71 | Unclassified: Metabolism |
| Bcf_03495 | Undecaprenyl-diphosphatase | -3.70 | Glycan biosynthesis and metabolism |
| Bcf_17870 | Ribonucleotide reductase of class III (anaerobic), large subunit | -3.68 | Nucleotide metabolism |
| Bcf_08420 | Flagellar biosynthesis protein FliR | -3.67 | Cell motility |
| Bcf_02135 | Anaerobic C4-dicarboxylate transporter | -3.63 | Transporters |
| Bcf_13370 | RNA polymerase sigma factor SigX | -3.62 | Transcription machinery |
| Bcf_21800 | Putative O-methyltransferase | -3.59 | Not found |
| Bcf_14080 | Drug resistance transporter, EmrB/QacA family | -3.56 | Transporters |
| Bcf_12400 | Exosporium protein D | -3.54 | Unclassified: Cell growth |
| Bcf_23930 | ATP synthase protein I | -3.54 | Photosystem and electron transport system |
| Bcf_11990 | Chloramphenicol acetyltransferase | -3.53 | Antimicrobial resistance genes |
| Bcf_26355 | Transmembrane protein | -3.50 | Not Included in Pathway or Brite |
| Bcf_13445 | Lipoprotein | -3.50 | Not found |
| Bcf_26075 | S-adenosylmethionine decarboxylase | -3.50 | Amino acid metabolism |
| Bcf_08200 | RNA-binding protein Hfq | -3.48 | Folding, sorting and degradation / Cellular community - prokaryotes |
| Bcf_08530 | LysR family transcriptional regulator | -3.46 | Transcription factors |
| Bcf_25970 | Cold shock protein CspC | -3.45 | Unclassified: Transcription |
| Bcf_13430 | Integral membrane protein | -3.44 | Unclassified: Structural proteins |
| Bcf_02205 | Major facilitator family transporter | -3.43 | Transporters |
| Bcf_26865 | Bacillolysin | -3.42 | Peptidases and inhibitors |
| Bcf_03980 | PTS system sucrose-specific transporter subunit IIB | -3.41 | Carbohydrate metabolism / Environmental Information Processing |
| Bcf_17965 | Spore coat protein M | -3.40 | Unclassified: Cell growth |
| Bcf_17730 | Spore germination protein GerSC | -3.40 | Not found |
| Bcf_12785 | Exosporium protein K | -3.40 | Not found |
| Bcf_24760 | ABC transporter ATP-binding protein | -3.39 | Transporters |
| Bcf_20940 | TetR family transcriptional regulator | -3.36 | Transcription factors |
| Bcf_03510 | DNA-binding protein | -3.36 | Not found |
| Bcf_06625 | Formate efflux transporter | -3.34 | Transporters |
| Bcf_08715 | Na+ driven multidrug efflux pump | -3.34 | Transporters |
| Bcf_03210 | Amino acid permease family protein | -3.34 | Transporters |
| Bcf_08390 | Flagellar motor switch protein FliN | -3.33 | Cell motility |
| Bcf_22020 | Forespore-specific protein | -3.32 | Not found |
| Bcf_04465 | Putative transcriptional regulator | -3.29 | Transcription factors |
| Bcf_20175 | Segregation and condensation protein B | -3.27 | Chromosome and associated proteins |
| Bcf_15520 | Carbonic anhydrase | -3.26 | Energy metabolism |
| Bcf_08290 | Flagellar basal-body rod protein FlgB | -3.24 | Cell motility |
| Bcf_21210 | 50S ribosomal protein L33 | -3.23 | Translation |
| Bcf_19815 | Glutaredoxin family protein | -3.22 | Not found |
| Bcf_18400 | Flagellar hook-length control protein FliK | -3.22 | Cell motility |
| Bcf_23805 | ABC transporter permease | -3.21 | Transporters |
| Bcf_11575 | Thioredoxin | -3.21 | Not found |
| Bcf_08435 | Flagellar biosynthesis protein FlhF | -3.19 | Bacterial motility proteins |
| Bcf_02765 | ABC transporter ATP-binding protein | -3.19 | Transporters |
| Bcf_07355 | Low-affinity inorganic phosphate transporter like protein | -3.17 | Unclassified: Transport |
| Bcf_22225 | Membrane Spanning Protein | -3.17 | Not found |
| Bcf_07240 | Peptidase, M23/M37 family | -3.16 | Peptidases and inhibitors |
| Bcf_02980 | GNAT family acetyltransferase | -3.15 | Amino acid metabolism |
| Bcf_03140 | Di-/tripeptide transporter | -3.15 | Unclassified: Transport |
| Bcf_17875 | Ribonucleotide reductase of class III (anaerobic), large subunit | -3.13 | Nucleotide metabolism |
| Bcf_03535 | Branched-chain amino acid transport system carrier protein | -3.13 | Unclassified: Transport |
| Bcf_02470 | Glycosyl transferase, group 2 family protein | -3.12 | Lipid metabolism |
| Bcf_06220 | Indole-3-glycerol phosphate synthase | -3.12 | Amino acid metabolism |
| Bcf_25265 | Two-component response regulator vanRB | -3.11 | Not found |
| Bcf_23730 | Rhodanese-like domain-containing protein | -3.10 | Not found |
| Bcf_05460 | TetR family transcriptional regulator | -3.09 | Transcription factors |
| Bcf_06225 | Phosphoribosylanthranilate isomerase | -3.07 | Amino acid metabolism |
| Bcf_27240 | Methyl-accepting chemotaxis protein | -3.07 | Bacterial motility proteins |
| Bcf_00255 | Small, acid-soluble spore protein | -3.07 | Unclassified: Cell growth |
| Bcf_17435 | Cold shock protein CspD | -3.06 | Unclassified: Transcription |
| Bcf_06285 | NADH oxidase | -3.05 | Not found |
| Bcf_07505 | Ferredoxin | -3.04 | Unclassified: Metabolism |
| Bcf_13450 | Spore germination protein YpeB | -3.04 | Unclassified: Cell growth |
| Bcf_19690 | Protein-glutamine gamma-glutamyltransferase | -3.04 | Unclassified: Metabolism |
| Bcf_11115 | Sodium-dependent transporter | -3.04 | Unclassified: Transport |
| Bcf_16330 | Thiol-activated cytolysin | -3.04 | Cellular community - prokaryotes |
| Bcf_02485 | Glycosyl transferase, group 2 family protein | -3.02 | Lipid metabolism |
| Bcf_02100 | Alpha-glucosidase | 3.03 | Carbohydrate metabolism |
| Bcf_05610 | AraC family transcriptional regulator | 3.04 | Transcription factors |
| Bcf_05640 | Peptidase, M48 family | 3.09 | Peptidases and inhibitors |
| Bcf_00235 | Ribonuclease M5 | 3.10 | Unclassified: Metabolism |
| Bcf_02620 | Potassium channel protein | 3.11 | Not found |
| Bcf_09840 | ABC transporter permease | 3.12 | Signal transduction |
| Bcf_25630 | Central glycolytic genes regulator | 3.12 | Transcription factors |
| Bcf_03300 | Glutamate transport membrane-spanning protein | 3.13 | Membrane transport |
| Bcf_13265 | GNAT family acetyltransferase | 3.13 | Amino acid metabolism |
| Bcf_23220 | Argininosuccinate synthase | 3.14 | Amino acid metabolism |
| Bcf_19450 | Cell division protein FtsI like / Peptidoglycan synthetase | 3.15 | Glycan biosynthesis and metabolism |
| Bcf_01980 | Fe3+-siderophore ABC transporter permease 2 component | 3.15 | Not found |
| Bcf_03290 | Glutamine ABC transporter substrate-binding protein | 3.16 | Membrane transport |
| Bcf_20270 | Anti-sigma B factor antagonist RsbV | 3.17 | Unclassified: Cell growth |
| Bcf_08640 | Cobalt-zinc-cadmium resistance protein CzcD | 3.19 | Transporters |
| Bcf_17405 | Vancomycin B-type resistance protein VanW | 3.19 | Signal transduction / Drug resistance: antimicrobial |
| Bcf_20360 | Stage II sporulation protein M (SpoIIM) | 3.20 | Unclassified: Cell growth |
| Bcf_20820 | Stage III sporulation protein AF | 3.21 | Unclassified: Cell growth |
| Bcf_02825 | Serine protein kinase (prkA protein), P-loop containing | 3.22 | Unclassified: Signaling proteins |
| Bcf_13350 | Pullulanase | 3.22 | Carbohydrate metabolism |
| Bcf_17790 | ECF-type sigma factor negative effector | 3.23 | Transcription machinery |
| Bcf_18125 | Hut operon positive regulatory protein | 3.23 | Other transcription factors |
| Bcf_25610 | 2,3-bisphosphoglycerate-independent phosphoglycerate mutase | 3.24 | Carbohydrate metabolism / Energy metabolism |
| Bcf_20640 | Glutamine transport ATP-binding protein GlnQ | 3.27 | Membrane transport |
| Bcf_20745 | DNA repair protein RecN | 3.31 | DNA repair and recombination proteins |
| Bcf_01985 | Fe3+-siderophore ABC transporter permease | 3.34 | Not found |
| Bcf_11305 | Iron(III) dicitrate transport system, periplasmic iron-binding protein FecB | 3.34 | Membrane transport |
| Bcf_18410 | tRNA delta(2)-isopentenylpyrophosphate transferase | 3.35 | Metabolism of terpenoids and polyketides |
| Bcf_04165 | ABC transporter permease | 3.36 | Membrane transport |
| Bcf_10620 | SpoVS-related protein, type 1 | 3.37 | Unclassified: Cell growth |
| Bcf_00880 | Polysaccharide deacetylase | 3.39 | Unclassified: Metabolism |
| Bcf_13645 | Glycine betaine ABC transport system, ATP- binding protein OpuAA | 3.42 | Membrane transport |
| Bcf_18425 | PTS system fructose-specific transporter subunit IIA | 3.46 | Carbohydrate metabolism |
| Bcf_20810 | Stage III sporulation protein AH | 3.47 | Unclassified: Cell growth |
| Bcf_21945 | Stage V sporulation protein B | 3.50 | Unclassified: Cell growth |
| Bcf_21410 | Stage IV sporulation protein | 3.53 | Unclassified: Cell growth |
| Bcf_09845 | Transcription state regulatory protein abrB | 3.55 | Transcription factors |
| Bcf_24170 | LPXTG-site transpeptidase family protein | 3.56 | Peptidases and inhibitors |
| Bcf_13160 | MutT/nudix family protein | 3.57 | DNA repair and recombination proteins |
| Bcf_27035 | Prespore specific transcriptional activator RsfA | 3.58 | Transcription factors |
| Bcf_07740 | Uracil-DNA glycosylase | 3.58 | Replication and repair |
| Bcf_03030 | Alanine dehydrogenase | 3.61 | Amino acid metabolism |
| Bcf_17740 | Chromosome segregation ATPase | 3.62 | Chromosome and associated proteins |
| Bcf_21995 | Substrate-binding family protein | 3.63 | Not found |
| Bcf_19945 | Maltose/maltodextrin ABC transporter permease MalG | 3.67 | Membrane transport |
| Bcf_11915 | Zinc transporter, ZIP family | 3.68 | Transporters |
| Bcf_17155 | Fe3+-siderophore ABC transporter permease 2 component | 3.74 | Not found |
| Bcf_20275 | D-alanyl-D-alanine carboxypeptidase | 3.76 | Glycan biosynthesis and metabolism |
| Bcf_03285 | Glutamate transport ATP-binding protein | 3.77 | Membrane transport |
| Bcf_26510 | Stage II sporulation protein D (SpoIID) | 3.77 | Unclassified: Cell growth |
| Bcf_10045 | Membrane protein involved in the export of O- antigen, teichoic acid lipoteichoic acids | 3.77 | Not found |
| Bcf_20830 | Stage III sporulation protein AD | 3.78 | Unclassified: Cell growth |
| Bcf_08690 | Class II fumarate hydratase | 3.78 | Carbohydrate metabolism / Energy metabolism |
| Bcf_01405 | Alanine racemase | 3.80 | Metabolism of other amino acids |
| Bcf_09575 | Cytochrome d ubiquinol oxidase subunit II | 3.81 | Energy metabolism / Signal transduction |
| Bcf_19970 | Maltose/maltodextrin transport ATP-binding protein MalK | 3.82 | Membrane transport |
| Bcf_09570 | Cytochrome d ubiquinol oxidase subunit I | 3.84 | Energy metabolism / Signal transduction |
| Bcf_22070 | ABC transporter permease | 3.87 | Transporters |
| Bcf_07640 | Stage IV sporulation protein A | 3.97 | Unclassified: Cell growth |
| Bcf_17710 | Formate dehydrogenase related protein | 3.97 | Carbohydrate metabolism / Energy metabolism |
| Bcf_07435 | D-alanyl-D-alanine carboxypeptidase | 3.97 | Glycan biosynthesis and metabolism |
| Bcf_24165 | DNA-binding response regulator | 3.99 | Two-component system |
| Bcf_22435 | Germination (Cortex hydrolysis) and sporulation protein GerM | 4.07 | Unclassified: Cell growth |
| Bcf_09765 | Acyl carrier protein associated with anthrachelin biosynthesis | 4.07 | Metabolism of terpenoids and polyketides |
| Bcf_14575 | Antibiotic biosynthesis monooxygenase domain- containing protein | 4.08 | Biosynthesis of other secondary metabolites |
| Bcf_12365 | Pyruvate decarboxylase | 4.10 | Carbohydrate metabolism |
| Bcf_20815 | Stage III sporulation protein AG | 4.13 | Unclassified: Cell growth |
| Bcf_07440 | Spore maturation protein A | 4.17 | Unclassified: Cell growth |
| Bcf_07445 | Spore maturation protein B | 4.19 | Unclassified: Cell growth |
| Bcf_09760 | Long-chain-fatty-acid--CoA ligase | 4.21 | Metabolism of terpenoids and polyketides |
| Bcf_22065 | ABC transporter permease | 4.22 | Transporters |
| Bcf_20555 | Glutamate N-acetyltransferase | 4.23 | Amino acid metabolism |
| Bcf_22125 | Stage IV sporulation protein FA (SpoIVFA) | 4.24 | Unclassified: Cell growth |
| Bcf_20650 | Amino acid ABC transporter substrate-binding protein | 4.35 | Membrane transport |
| Bcf_25550 | Petrobactin ABC transporter substrate-binding protein | 4.45 | Not found |
| Bcf_22740 | NPQTN specific sortase B | 4.48 | Peptidases and inhibitors |
| Bcf_19515 | Membrane protein | 4.51 | Not found |
| Bcf_20840 | Stage III sporulation protein AB | 4.59 | Unclassified: Cell growth |
| Bcf_12450 | Pyruvate decarboxylase | 4.62 | Carbohydrate metabolism |
| Bcf_22075 | ABC transporter ATP-binding protein | 4.67 | Transporters |
| Bcf_26490 | Stage II sporulation protein related to metaloproteases (SpoIIQ) | 4.90 | Unclassified: Cell growth |
| Bcf_20835 | Stage III sporulation protein AC | 4.98 | Unclassified: Cell growth |
| Bcf_20560 | N-acetyl-gamma-glutamyl-phosphate reductase | 5.05 | Amino acid metabolism |
| Bcf_20845 | Stage III sporulation protein AA | 5.08 | Unclassified: Cell growth |
| Bcf_19950 | Maltose/maltodextrin ABC transporter permease MalF | 5.12 | Membrane transport |
| Bcf_19240 | Cation-transporting ATPase | 5.15 | Unclassified: Metabolism |
| Bcf_17160 | Fe3+-siderophore ABC transporter permease | 5.22 | Not found |
| Bcf_26015 | UDP-glucose dehydrogenase | 5.23 | Carbohydrate metabolism / Glycan biosynthesis and metabolism |
| Bcf_04035 | Putative nitroreductase family protein | 5.26 | Not found |
| Bcf_11890 | ABC transporter permease | 5.29 | Transporters |
| Bcf_18510 | Trilactone hydrolase (bacillibactin) siderophore | 5.39 | Poorly characterized |
| Bcf_09755 | Anthrachelin biosynthesis protein AsbB / Siderophore synthetase superfamily, group C / Siderophore synthetase component, ligase | 5.56 | Metabolism of terpenoids and polyketides |
| Bcf_12455 | Pyruvate decarboxylase | 5.62 | Carbohydrate metabolism |
| Bcf_11780 | 2,3-dihydro-2,3-dihydroxybenzoate dehydrogenase | 5.64 | Metabolism of terpenoids and polyketides |
| Bcf_20825 | Stage III sporulation protein AE | 5.66 | Unclassified: Cell growth |
| Bcf_09750 | Anthrachelin biosynthesis protein AsbA / Siderophore synthetase superfamily, group A / Siderophore synthetase large component, acetyltransferase | 5.99 | Metabolism of terpenoids and polyketides |
| Bcf_11885 | ABC transporter ATP-binding protein | 6.20 | Transporters |
| Bcf_22745 | Heme transporter analogous to IsdDEF, ATP- binding protein | 6.35 | Not found |
| Bcf_19955 | Maltose/maltodextrinABC transporter substrate-binding protein MalE | 6.37 | Membrane transport |
| Bcf_22755 | Heme transporter IsdDEF, lipoprotein IsdE | 6.49 | Transporters |
| Bcf_18515 | Fe-bacillibactin uptake system FeuD | 6.68 | Transporters |
| Bcf_18525 | Fe-bacillibactin uptake system FeuB | 6.71 | Transporters |
| Bcf_03370 | Oligopeptide ABC transporter substrate-binding protein OppA | 6.87 | Membrane transport / Cellular community - prokaryotes |
| Bcf_22750 | Heme transporter IsdDEF, permease component IsdF | 7.15 | Transporters |
| Bcf_11815 | 4'-phosphopantetheinyl transferase | 7.31 | Metabolism of cofactors and vitamins |
| Bcf_11795 | Isochorismatase | 7.40 | Metabolism of terpenoids and polyketides |
| Bcf_16240 | Internalin | 7.68 | Infectious disease: bacterial |
| Bcf_13340 | Bacitracin transport permease protein | 7.82 | Membrane transport / Signal transduction |
| Bcf_11810 | Polymyxin transporter PmxC | 7.94 | Not found |
| Bcf_05665 | S-layer protein | 7.98 | Unclassified: Structural proteins |
| Bcf_18520 | Fe-bacillibactin uptake system FeuC | 8.14 | Transporters |
| Bcf_06735 | Internalin | 8.48 | Infectious disease: bacterial |
| Bcf_11800 | Siderophore biosynthesis non-ribosomal peptide synthetase / Bacillibactin synthetase component F | 8.52 | Metabolism of terpenoids and polyketides |
| Bcf_11805 | Polymyxin synthetase PmxB | 8.54 | Biosynthesis of other secondary metabolites |
| Bcf_18535 | Fe-bacillibactin uptake system FeuA | 8.55 | Transporters |
| Bcf_11785 | Isochorismate synthase | 8.58 | Metabolism of cofactors and vitamins / Metabolism of terpenoids and polyketides |
| Bcf_21720 | Iron compound ABC transporter ATP-binding protein | 9.01 | Transporters |
| Bcf_22760 | Cell surface protein IsdA | 9.31 | Infectious disease: bacterial |
| Bcf_22765 | NPQTN cell wall anchored protein IsdC | 9.58 | Transporters |
| Bcf_06965 | Flavodoxin | 10.01 | Unclassified: Metabolism |
| Bcf_11790 | 2,3-dihydroxybenzoate-AMP ligase | 10.31 | Metabolism of terpenoids and polyketides |
| Bcf_21715 | Ankyrin repeat domain protein | 10.67 | Membrane trafficking |
| Bcf_21730 | Heme transporter IsdDEF, lipoprotein IsdE | 10.90 | Transporters |
| Bcf_17445 | Flavodoxin | 12.14 | Unclassified: Metabolism |
| Bcf_21725 | Fe3+-siderophore ABC transporter permease | 12.23 | Transporters |

**References**

Beecher, D. J. & Macmillan, J. D. (1991). Characterization of the components of hemolysin BL from *Bacillus cereus*. *Infect. Immun.* 59(5), 1778-1784

Jeßberger, N., Krey, V. M., Rademacher, C., Böhm, M. E., Mohr, A. K., Ehling-Schulz, M., Scherer, S. & Märtlbauer, E. (2015). From genome to toxicity: a combinatory approach highlights the complexity of enterotoxin production in *Bacillus cereus*. *Front. Microbiol.* 6, 560. https://doi.org/10.3389/fmicb.2015.00560

Jugert, C. S., Didier, A., Plötz, M., & Jessberger, N. (2023). Strain-specific antimicrobial activity of lactoferrin-based food supplements. *J. Food Prot.* In press. doi:10.1016/j.jfp.2023.100153

Lund, T. & Granum, P.E. (1996). Characterisation of a non-haemolytic enterotoxin complex from *Bacillus cereus* isolated after a foodborne outbreak. *FEMS Microbiol. Lett.* 141, 151–156. https://doi.org/10.1111/j.1574-6968.1996.tb08377.x

Schwenk, V., Riegg, J., Lacroix, M., Märtlbauer, E., & Jessberger, N. (2020). Enteropathogenic potential of *Bacillus thuringiensis* isolates from soil, animals, food and biopesticides. *Foods* 9(10), 1484. doi:10.3390/foods9101484
